# Malaria Case Detection Among Mobile Populations and Migrant Workers in Myanmar: Comparison of 3 Service Delivery Approaches

**DOI:** 10.9745/GHSP-D-17-00318

**Published:** 2018-06-27

**Authors:** Soy Ty Kheang, May Aung Lin, Saw Lwin, Ye Hein Naing, Phyo Yarzar, Neeraj Kak, Taylor Price

**Affiliations:** a University Research Co., LLC, Phnom Penh, Cambodia.; b University Research Co., LLC, Yangon, Myanmar.; c University Research Co., LLC, Chevy Chase, MD, USA.

## Abstract

**Background:**

Mobile populations and migrant workers are a key population to containing the spread of artemisinin-resistant malaria found in the border areas between Cambodia, Myanmar, and Thailand. Migrants often have limited knowledge of public health, including malaria, services in the area, and many seek care from unregulated, private vendors.

**Methods:**

Between October 2012 and August 2016, we implemented malaria case finding and treatment in Tanintharyi Region, Kayin State, and Rakhine State of Myanmar through 3 entry points: village malaria workers (VMWs), mobile malaria clinics, and screening points. A total of 1,000 VMWs provided passive case detection and treatment services to residents in malaria-endemic villages. Active case finding through mobile malaria clinics was conducted by staff in 354 remote villages and work sites, where regular monitoring and supervision of VMWs would be difficult to maintain. Malaria screening points were a hybrid combination of active and passive case finding in which screening points were set up at fixed locations in Tanintharyi Region and Kayin State, such as bus stops, ferry docks, or informal border crossing points, and migrants entering into or departing from endemic areas could voluntarily receive malaria testing and treatment. Using routine monitoring data, we assessed and compared the malaria positive rate—the number of positive malaria cases out of those tested—across the 3 approaches as an indication of the programmatic effectiveness in identifying malaria cases in the population. Most testing was conducted with rapid diagnostic tests.

**Results:**

Mobile teams (169,859) and VMWs (157,048) tested a higher number of community members than screening points (3,676) as they covered a wider geographical area. However, the malaria positive rate was higher among VMWs (7.29%) and screening points (7.10%) than mobile teams (2.64%). VMWs were located in hard-to-access areas that have higher malaria prevalence and are difficult to reach by vehicle while screening points specifically targeted mobile populations and migrant workers. Mobile teams also screened non-fever patients during their visits, which may explain their lower malaria positive rate.

**Conclusions:**

A combination of malaria testing approaches helps achieve both maximum reach and high case finding as it allows access to a range of migrant communities and provides an opportunity for continuity of service delivery as the migrants travel to their destinations.

## Abstract (precis)

In 3 regions of Myanmar, village malaria workers (VMWs) and mobile teams tested a higher number of people than strategically placed fixed screening points at border crossings, but VMWs and screening points yielded higher malaria positive rates. We recommend using a combination of these approaches in the Greater Mekong Subregion for such populations depending on the strategic approach of the program.

## BACKGROUND

Mobile populations and migrant workers who move from their permanent residence to malaria-endemic areas for work or other purposes are a key population to containing the spread of artemisinin-resistant malaria found in the border areas between Cambodia, Myanmar, and Thailand.^1^ There are an estimated 2 million Burmese migrants and 248,000 Cambodian migrants working in Thailand, the main recipient country in the Greater Mekong Subregion.^2^

There is also significant internal migration in the countries. For instance, it is known that most migrants in Cambodia are internal,^3^ and while there is little information on migration within Myanmar, it is estimated to be very high.^4^ A study conducted in Myanmar in 2015 by the International Labour Organization found that a greater percentage of internal labor migrants migrate for work across states or regions within the country rather than within their own states/regions (62% vs. 38%, respectively).^5^ However, this varied across Myanmar. In addition, those migrating from a rural area were likely to migrate to another rural area. In Magway and Ayeyarwady regions, the majority of migrants are men (66% and 60%, respectively) and about 80% are between the ages of 11 and 30 years.^6^ New to the community, they often have limited knowledge of public health services in the area, including how to prevent malaria, and many seek care from unregulated, private vendors.^7^

There are, however, differences between migrant communities. A study by Wangroongsarb et al. found significant differences between long-term (over 6 months) and short-term Burmese and Cambodian migrants living in Thailand's border areas. Most long-term migrants had heard of malaria, but only about half of short-term migrants had. Short-term migrants were also more likely than long-term migrants not to know any malaria symptoms or preventive methods. Yet a majority (over 86% for all groups) had slept under a bed net the previous night, in accordance with the cultural norms in the Greater Mekong Subregion.^8^

The President's Malaria Initiative (PMI) and the United States Agency for International Development (USAID) Control and Prevention of Malaria (CAP-Malaria) Project, implemented between October 2012 and August 2016, worked in border areas between Cambodia, Myanmar, and Thailand to contain the spread of multidrug resistant *P. falciparum* malaria in the Greater Mekong, home to large numbers of mobile populations and migrant workers (Figure). CAP-Malaria worked in collaboration with Myanmar's National Malaria Control Program (NMCP) and ethnic health organizations in project areas.

**FIGURE:**
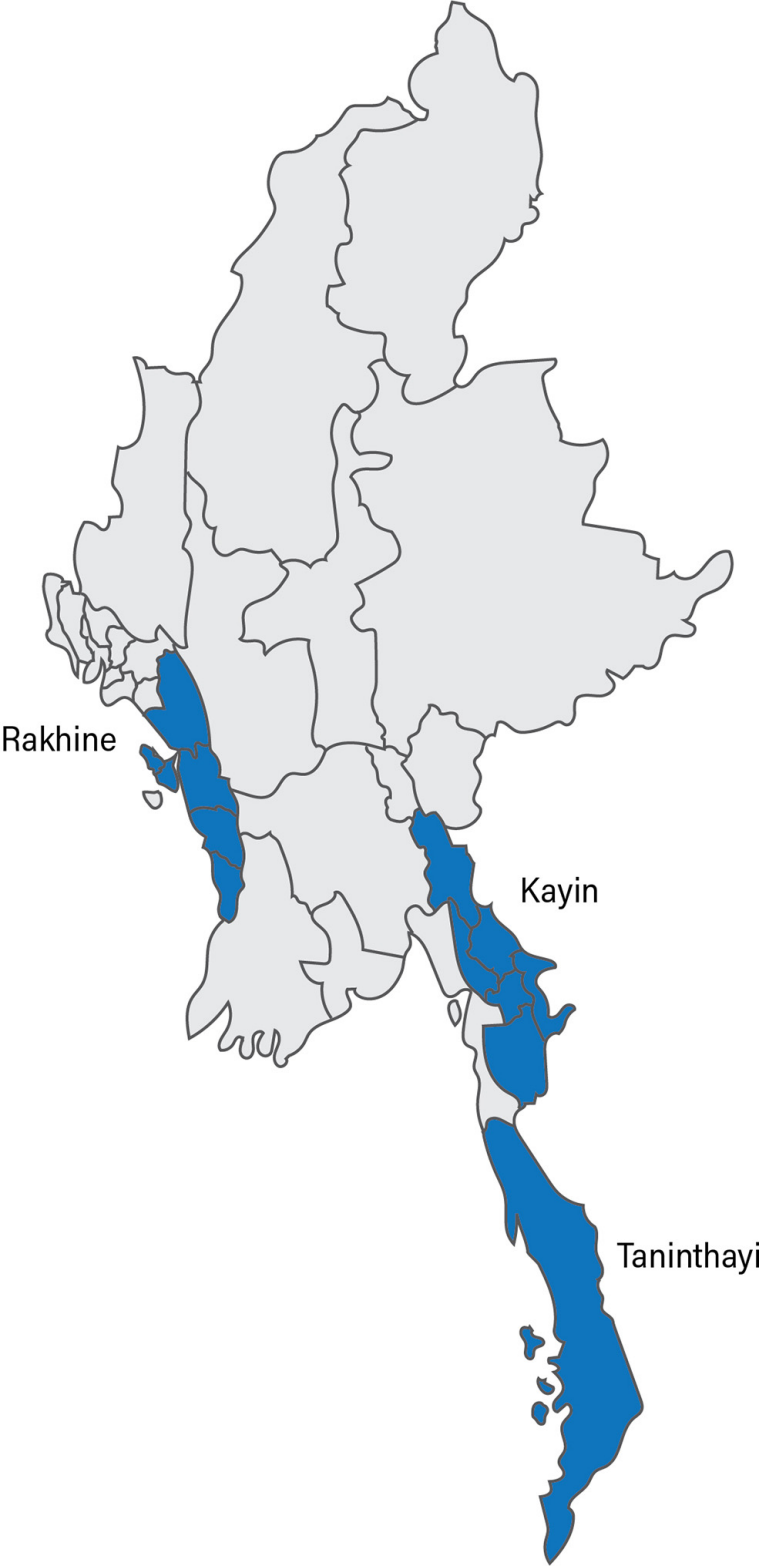
Areas Covered by the CAP-Malaria Project, October 2012–March 2015 Abbreviation: CAP-Malaria, Control and Prevention of Malaria. The project worked in 10 townships in Tanintharyi Region, 7 townships in southern Rakhine State, and 6 townships in Kayin State.

The CAP-Malaria Project worked in border areas in the Greater Mekong, home to large numbers of mobile populations and migrant workers, to contain the spread of multidrug resistant malaria.

Myanmar's *National Strategic Plan: Intensifying Malaria Control and Accelerating Progress Towards Malaria Elimination 2016–2020* mentions 2 types of populations: static and migrant.^9^ Static populations are those who live and work in their villages while migrants and mobile populations include seasonal agricultural laborers, defense services, nonstate actors, and forest workers. The plan identifies 3 key entry points for malaria service delivery for migrants who are scattered in remote areas in Myanmar: community-based village malaria workers (VMWs), mobile malaria clinics, and screening points. The purpose of this article is to describe the CAP-Malaria Project's activities using these 3 approaches and compare the malaria positive rate (number of positive cases out of those tested) among the 3 approaches to assess their effectiveness in malaria case detection.

## METHODS

### Intervention

CAP-Malaria's main approach was the establishment of VMW networks to fill service gaps in target areas. Mobile malaria clinics were also organized at least 3 times each year (especially during pre- and post-monsoon season, which lasts from May/June to early October) in very remote areas with a high malaria burden and/or in areas not covered by VMWs or private providers to conduct active case detection and management. Finally, the project established screening points at common entry points for migrants and mobile populations.

### Village Malaria Workers

Because malaria prevalence is often highest in the most remote locations, and as interpersonal communication was found to be the preferred source of malaria information in a CAP-Malaria survey, CAP-Malaria supported at least 1 VMW in each of the target villages that were geographically hard-to-reach and had high malaria case load/transmission, no appointed public health staff, and no other malaria service providers. The VMWs were recruited from their own communities with the help of village chiefs. From October 2012 to March 2015, 1,000 VMWs in total provided malaria prevention and control services in 23 townships in Tanintharyi Region and Kayin and Rakhine States.

Between 2012 and 2015, 1,000 village malaria workers provided malaria prevention and control services in 23 townships in Myanmar.

VMWs have a strong network with long-term and temporary community residents, and are familiar with local health services. They were trained to provide their respective communities malaria education; help migrants recognize malaria symptoms; diagnose malaria with rapid diagnostic tests; treat simple malaria according to national malaria treatment guidelines and monitor treatment adherence; and refer severe cases (with no initial treatment) and pregnant women to health centers. Support and supervision was provided during visits by mobile malaria clinics or at monthly township-level meetings to monitor existing or potential diagnostic and treatment stock-outs for provision of continuous malaria services. During the monthly meetings, basic health staff replenished VMW supplies, collected reports, discussed challenges and solutions, and provided refresher training.

### Mobile Malaria Clinics

For migrant communities in remote villages or work sites that could be accessed by motorbike or vehicle, CAP-Malaria introduced mobile malaria clinics to provide screening and treatment of malaria cases. Criteria for communities to be eligible for mobile malaria clinics included: no health facility or volunteer services, recruitment of VMWs impractical due to high costs and inability for them to attend monthly meetings and regularly submit reports because of access issues, or a high malaria burden. Mobile malaria clinics were staffed by a doctor, a microscopist, a health worker, and, when needed, an interpreter. Each mobile clinic served approximately 30 zones or villages in its assigned area. The timing of the mobile clinic's visit was coordinated with the VMW or other local contact who promoted the visit in the community, and village chiefs provided community mobilization. The mobile clinics provided support to the VMWs, replenishing supplies and answering questions.

**Figure:**
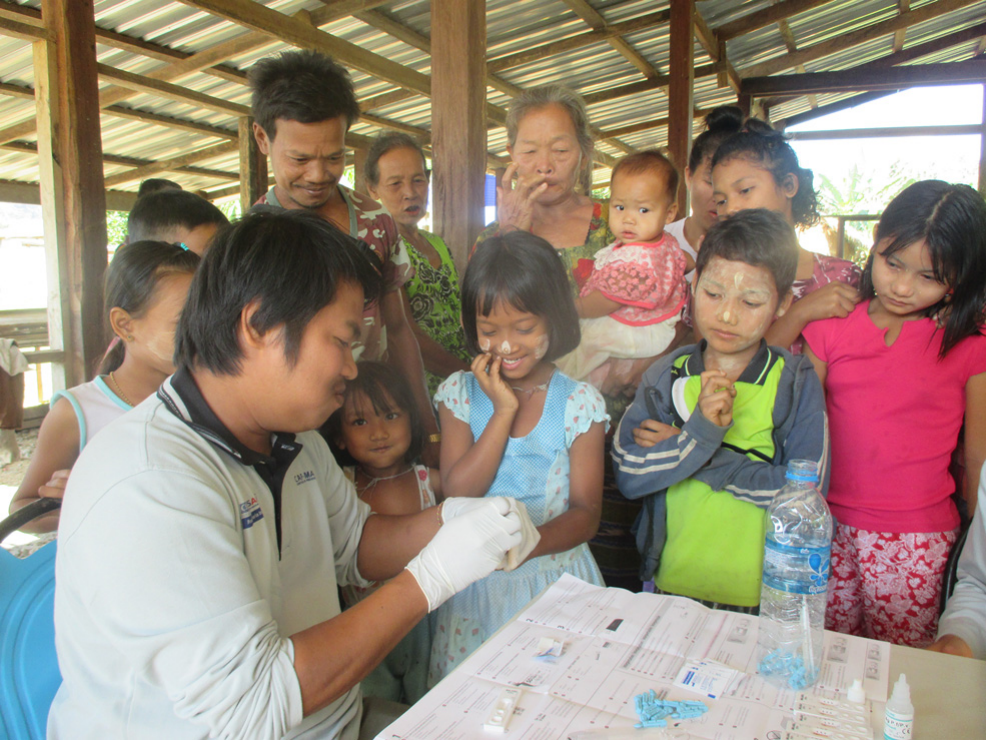
Mobile clinic staff conduct active case detection of malaria at Hte Hta village, Dawei in Taintharyi Region of Myanmar. © 2017 University Research Co., LLC.

From October 2012 to March 2015, mobile clinics visited 354 villages/work sites in 23 townships in Tanintharyi Region and Kayin and Rakhine States. At the village level, the mobile clinics raised communities' awareness about malaria prevention and the importance of early diagnosis and prompt and adequate treatment. They also offered testing services to anyone in the target community.

Mobile clinics visited 354 villages and work sites in remote areas.

### Screening Points

The project set up malaria screening points at key fixed locations aimed to reach migrants while traveling between sites or during entry into or departure from endemic areas, such as bus stations or jetty terminals. For example, each month thousands pass through the Myoma Jetty and Bus Terminal in Kawthoung Township, a major departure area for Burmese migrants. Screening points were decided in discussion with Township Medical Officers. Migrants could voluntarily have their temperature checked, be tested for malaria with a rapid diagnostic test, and receive treatment if necessary. Migrants were also provided with information on malaria risks, services available, and prevention methods. Screening points were established at bus and boat terminals in Bokpyin and Kawthoung townships in Tanintharyi Region and Hpa-an, Hlaingbwe, Kawkareik, and Myawaddy townships in Kayin State. All testing activities were on a volunteer basis and provided free of charge.

### Data Collection

The malaria positive rate, or the number of positive cases out of those tested, provides a good indication of the programmatic effectiveness in identifying malaria cases in a population. The number of cases tested and diagnosed through each of the 3 approaches mentioned above (VMWs, mobile malaria clinics, screening points) was monitored between October 2012 and March 2015 in Tanintharyi Region and Kayin and Rakhine States. Data were collected from the CAP-Malaria summary database after validation against source documents (e.g., VMW and health facility registers) by CAP-Malaria staff. Most testing was done by a rapid diagnostic test, with microscopy used only in health facilities implementing a strong quality assurance/quality control system. Privacy and confidentiality of personal information were maintained throughout data collection and analysis.

## RESULTS

Between October 2012 and March 2015, CAP-Malaria tested more than 330,000 people and provided appropriate treatment to more than 16,000 people who had been diagnosed with malaria (Table).

**TABLE.** Malaria Positive Rate by Service Delivery Approach in Tanintharyi Region and Kayin and Rakhine States, Myanmar, October 2012–March 2015

Approach	Total Tested				Positive Cases				MPR
	FY2012	FY2013	FY2014 (Q1–Q2)	Total	FY2012	FY2013	FY2014 (Q1–Q2)	Total	
Mobile malaria clinics	41,550	61,725	66,584	169,859	1,748	1,082	1,649	4,479	2.64%
VMWs	21,978	86,316	48,754	157,048	3,290	5,495	2,669	11,454	7.29%
Screening points	884	1,953	839	3,676	116	119	26	261	7.10%
Total	64,412	149,994	116,177	330,583	5,154	6,696	4,344	16,194	4.90%

Abbreviations: FY, fiscal year (October to September); MPR, malaria positive rate; Q, quarter; VMWs, village malaria workers.

Mobile teams were able to test a higher number of community members as they covered a wider geographical area providing active case detection. The mobile teams also screened non-fever patients during their visits. This may explain the relatively low malaria positive rate of 2.64%.

VMWs were an ongoing presence in the hard-to-access areas that have higher malaria prevalence and are difficult to reach by vehicle. VMWs provided passive case detection to patients presenting with fever or symptoms. They tested nearly as many people as mobile malaria clinics (157,048 vs. 169,859, respectively) but had a higher malaria positive rate, at 7.29%.

Village malaria workers tested nearly as many people as mobile clinics but had a higher malaria positive rate.

Screening points specifically targeted mobile populations and migrant workers. Although malaria screening points screened the fewest number of people (3,676 total), they yielded a similar malaria positive rate (7.10%) as VMWs, as these approaches screened suspected patients who presented with fever or symptoms.

## DISCUSSION

Effective malaria control among migrant workers and mobile populations requires a combination of approaches for 2 reasons: (1) there are differences between migrant groups in terms of knowledge, health-seeking behavior, and access to services, and (2) the combination of approaches maximizes the opportunities for migrants to obtain information and services while they travel and at their destination community. The 3 service delivery approaches used by the CAP-Malaria project have their advantages and disadvantages in terms of operation and cost.

**Figure:**
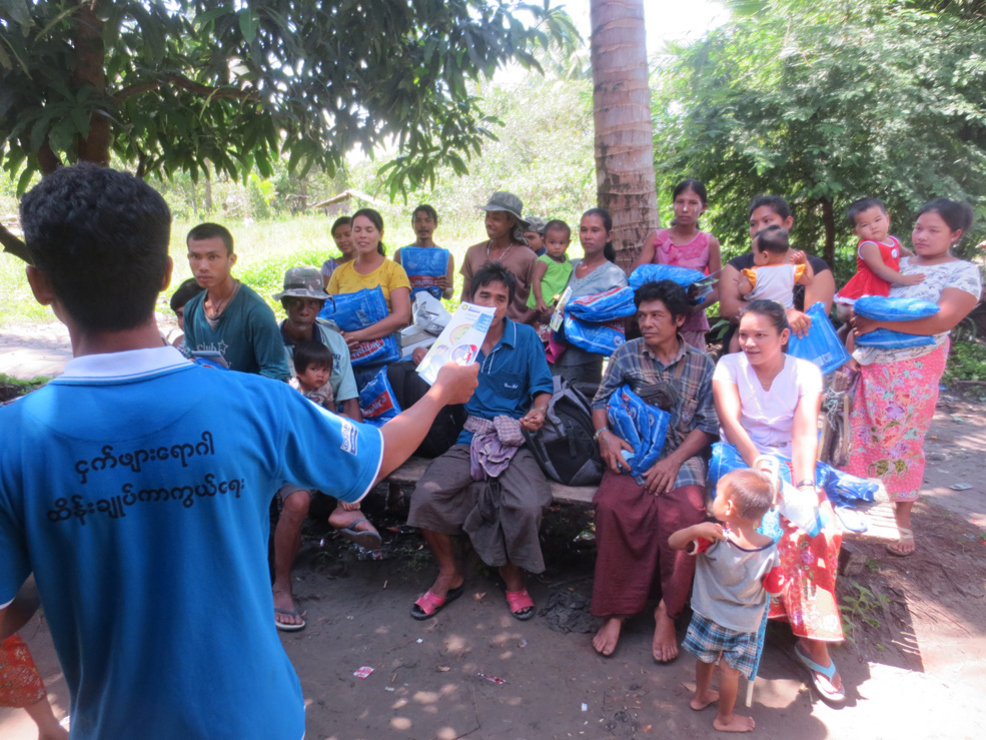
A village malaria worker provides health education to community members in Htaung Phee village in Bokpyin Township, Tanintharyi Region of Myanmar. © 2015 University Research Co., LLC.

The use of mobile teams can be expensive depending on frequency, distance, and remoteness of the areas covered. However, the more aggressive nature of case finding allows the program to access more people and identify cases that may normally be missed through routine passive case detection. Mobile teams should be provided as a complementary approach to health facility-based and community-based malaria testing and treatment services. Updated malaria information in the local context should be used to plan and implement mobile team activities to ensure higher malaria positive rate and for cost-control measures.

The use of VMWs requires a strong monitoring and supervision component to ensure quality of uninterrupted services by VMWs in the community. Operational costs are lower when the monitoring and supervision is integrated into local health systems and rural health staff are engaged as part of monitoring and supervision of VMWs. While this approach is effective in treating uncomplicated malaria, strong linkages and referral systems must be set up to refer complicated malaria cases. Village-based stratification of malaria information is important to effectively allocate resources.

Screening points, strategically located in areas highly frequented by migrant communities, combine active and passive case detection. The location and timing of screening points, and the criteria for screening, are important factors in the resulting malaria positive rate. Updated information and continuous evaluation of the results will help to better target resources to improve the malaria positive rate using this approach.

The most efficient approach for case finding appears to be through VMWs since they provide quality services with minimal resources required. However, VMWs have only limited reach in a population as they provide services only within their community. In comparison, mobile clinics identify fewer cases but are able to reach more people with malaria services and malaria information with wider geographical coverage. Scheduled mobile clinics contribute to sustain VMWs' activities in remote villages and work sites by replenishing supplies and providing supervision. While screening points resulted in a fewer number of people being tested, they nevertheless identified a large number of malaria cases by specifically targeting mobile populations and migrant workers when routine monitoring was not efficient. Screening points also raise awareness of malaria among travelers, preparing them to recognize malaria symptoms and seek care more quickly in their destination community.^4^ Quality data from these approaches is important to stratify villages and high-risk areas. Village-based stratification of malaria information is important to effectively allocate resources.

Screening points identified a large number of cases by specifically targeting mobile populations and migrant workers.

## CONCLUSION

As Myanmar and other countries move toward pre-elimination/elimination, malaria will begin to cluster among certain high-risk groups, including migrants and mobile populations. A combination of program approaches helps increase testing among high-risk populations and achieve high case finding rates, which will be critical to achieve elimination.

## Notes

**Peer Reviewed**

**Funding:** This activity was funded by the President's Malaria Initiative (PMI) and United States Agency for International Development (USAID) through the Control and Prevention of Malaria (CAP-Malaria) project (Agreement Number: AID-486-A-12-00001) implemented by University Research Co., LLC.

**First Published Online:** June 6, 2018
